# The dosimetric effect of inhomogeneity correction in dynamic conformal arc stereotactic body radiation therapy for lung tumors

**DOI:** 10.1120/jacmp.v7i2.2236

**Published:** 2006-05-25

**Authors:** Brian Kavanagh, Meisong Ding, Tracey Schefter, Kelly Stuhr, Francis Newman

**Affiliations:** ^1^ Department of Radiation Oncology University of Colorado Aurora Colorado U.S.A.

**Keywords:** stereotactic body radiation therapy (SBRT), inhomogeneity correction, lung tumors, equivalent uniform dose

## Abstract

For patients treated with lung stereotactic body radiation therapy (SBRT) using dynamic conformal arcs, the influence of inhomogeneity correction (IC) on normal tissue and tumor dosimetry was studied. For the same numbers of monitor units, the planning target volume equivalent uniform doses calculated without path‐length IC were lower than those calculated with IC (mean difference 18%, range 1% to 34%; p<0.0001). Normal lung dose differences were of the same magnitude in the opposite direction. In reports of SBRT, it will be helpful to maintain clear communication about the type of IC used to avoid future uncertainties about true normal tissue tolerance and tumor dose‐response relationships.

PACS numbers: 87.50.Gi, 87.53.Bn, 87.53.Kn

## I. INTRODUCTION

Stereotactic body radiation therapy (SBRT) is the administration of high doses of radiation in a hypofractionated schedule, with the goal of eradicating one or more extracranial tumors. The American Society of Therapeutic Radiology and Oncology and the American College of Radiology have published guidelines for the overall process of SBRT,^(1)^ but there is no universal standard method of tissue inhomogeneity correction (IC) for SBRT. Because SBRT may be given using a variety of techniques, there is reason to question how influential IC is for any given method of treatment. Furthermore, since large fractions are used, systematic discrepancies have greater biological impact for SBRT than conventionally fractionated radiation therapy given the expected nonlinear impact on biologically equivalent dose. IC‐related dose discrepancies could emerge for any application of SBRT, depending on the amount of tissue inhomogeneity within the volumes through which the beams transit. However, the issue is particularly relevant for lung SBRT, where beams frequently pass through extensive regions of low‐density lung parenchyma. Here, we evaluate the impact of IC on the dosimetry of SBRT for lung tumors treated in a prospective Phase I trial of SBRT for lung metastases using dynamic conformal arcs.

## II. METHODS

The protocol was approved by the Institutional Review Board, and clinical details are reported elsewhere.^(2)^ Key eligibility criteria included one to three lung metastases from a solid tumor and maximum cumulative tumor diameter <7cm. Twelve patients enrolled. Eight patients had more than one lung lesion, for a total of 21 tumors treated. Patients were immobilized during simulation and treatment using a custom body mold, and respiratory control was achieved using a breath‐holding technique or abdominal compression. Each gross tumor volume (GTV) was expanded 5 mm to 7 mm radially and 10 mm to 15 mm cranio‐caudally to create a planning tumor volume (PTV). The planning CT scanner used for all patients analyzed had been calibrated previously and was not recalibrated at any time during the course of the study. Intravenous contrast material was not used for any of the planning scans, since the lesions to be treated were peripheral nodules that were readily separable from mediastinal structures. All patients had had recent diagnostic CT scans with contrast prior to the planning CT scan, but never on the same day, so no residual contrast material was ever noted on the planning CT scans.

All treatment was given using dynamic conformal arcs planned with dedicated software (BrainScan®; BrainLAB AG). The software has a built‐in path‐length correction IC algorithm, and doses given to patients were calculated using this algorithm. The 21 GTVs ranged from 0.53 cm^3^ to 39.8 cm^3^, and the PTVs ranged from 6.4 cm^3^ to 123 cm^3^. Dose escalation proceeded smoothly, and the PTV dose was escalated safely from 48 Gy to 60 Gy/3 fractions.

**Figure 1:**
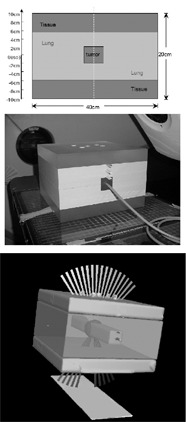
The lung phantom used for evaluating the inhomogeneity correction algorithm. The phantom allowed dose measurement via one of several measurement access points: at the isocenter, at the tumor—lung interface, within the lung‐equivalent material, and at the lung—tissue interface. Top panel: cross‐sectional sketch. The depth was 40 cm. Tissue‐like acrylic layers (density $1.0\;g/{\text{cm}}^{3}$) of 4 cm thickness surround lung‐equivalent polyurethane foam $\left(0.32\;g/{\text{cm}}^{3}\right)$, in the center of which is a tumor‐like acrylic section $\left(1.0\;g/{\text{cm}}^{3}\right)$. Center panel: photograph of the phantom with the ion chamber placed in the isocenter. Lower panel: schematic representation of the dynamic conformal arcs used to simulate an SBRT treatment.

To evaluate the path‐length IC algorithm used, ionization chamber measurements were taken in an inhomogeneous lung phantom (Fig. 1). A multiple dynamic conformal arc plan was used to simulate a typical SBRT treatment. Virtual PTVs of 3‐cm or 6‐cm diameter were targeted by three arcs with gantry rotations from 150° to 170°, 190° to 220°, and 320° to 40°, and with relative weights of 0.25, 0.25, and 0.5, respectively. The doses given to patients (GTV equivalent uniform dose (EUD), PTV EUD, $V_{15\%},\;V_{30\%}$ and $V_{50\%}$) were first calculated with path‐length IC. Here, $V_{15\%},\;V_{30\%}$, and $V_{50\%}$ refer to the percent of uninvolved lung that received 15%, 30%, or 50% of the prescription dose, respectively. The number of monitor units (MUs) per arc was recorded. The same parameters were then recalculated with the same MUs and arcs but *without* path‐length IC. EUDs were calculated according to established methods.^(^ ^3^ ^,^ ^4^ ^)^ The use of EUD for plan quality evaluations has been critiqued,^(5)^ but here EUD is simply used as an index for comparing dose.

It should be noted that within the software used, the doses given via arcs are calculated at finite 10° intervals, where the instantaneous multileaf collimator (MLC) position is considered as if it were a static beam. However, during treatment there is continuous linear MLC position adjustment. It is possible that additional dosimetric accuracy might be achieved if smaller arc increments were used (or, ideally, some means of modeling MLC motion continuously). While there is no *a priori* reason to expect that the percent discrepancy between the 10° incremental beam approximation and true dose delivered via arcs is influenced by IC, a complete answer to this question is beyond the scope of the present work. There is reason to expect that any dosimetric differences from the use of smaller arc increments within the calculation would be small for a somewhat similar clinical consideration, namely, intensity‐modulated arc therapy. Li and colleagues explored whether increments of 5° would add computational accuracy beyond what is achieved with 10° increments and found no significant difference.^(6)^

## III. RESULTS

For an isocenter dose of 24 Gy, the differences in phantom isocenter dose between measured and IC‐calculated doses were 0.3% and 2.3% for the 3‐cm and 6‐cm PTVs, respectively. In lung‐equivalent material, differences were 1.9% to 5.3%. At lung—tissue interfaces the differences between measured and calculated doses averaged 2.2% but ranged from −7% to +7%. The variability here was likely due at least partly to steep dose gradients.

For the same numbers of MUs, the EUDs calculated without IC were significantly lower than what was calculated with IC. The GTV EUD calculations differed by an average of 15% (range 0% to 26%), and the PTV EUD calculations differed by an average of 18% (range 1% to 34%; p<0.0001 by the paired *t*‐test for both comparisons). The percent difference in PTV EUD was not correlated with the tumor volume. Normal lung dose differences were of opposite direction to tumor dose comparisons. The $V_{15\%},\;V_{30\%}$, and $V_{50\%}$ calculated with IC were *lower*than the same without IC by an average of 13%, 15%, and 18%, respectively.

**Figure 2:**
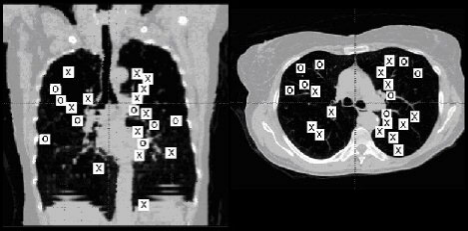
Coronal (left) and axial (right) composite locations of planning target volumes treated. Those marked with an X are lesions for which recalculation of the equivalent uniform dose without inhomogeneous correction (IC) differed by more than 15% from the value obtained with IC; for those marked with an O, the difference was less than 15%. See Results section for additional explanation.

To assess the effect of tumor location upon the IC effect, the PTV locations were converted into equivalent locations on a representative patient's planning CT images by maintaining the same proportionate distance from the carina to the periphery of the lung along a line segment oriented at the same angle in this figure as on the original images (Fig. 2). It should be noted that differences in thoracic contours cause slight distortion between images of the original PTV and the assigned location in the composite representation, limiting the analysis somewhat. Nevertheless, as shown in Fig. 2, tumors for which there was a higher discrepancy in dose calculation with versus without IC were located more centrally within the lung.

## IV. DISCUSSION

Results of the present study illustrate that during dynamic conformal arc SBRT for lung tumors, there are notable and significant differences in both tumor and normal lung dosimetry between calculations made with IC and those made without IC. The mean difference between GTV and PTV EUDs calculated with IC versus without IC exceeded the difference noted between doses calculated using the pencil beam (PB) algorithm applied and doses measured in a lung phantom.

The fact that IC tended to make a larger PTV dose difference for a more centrally located lesion, as shown in Fig. 2, is plausible when it is considered that for central lesions, a greater volume of lung tissue is traversed by the beam. The observation that the use of IC had an effect on normal lung dosimetric parameters that was opposite the effect upon PTV doses is likewise believable: the IC algorithm provides a more realistic assumption of reduced absorption within lung tissue, whereas without IC there is an assumption of radiation dose absorption within lung tissue at the same rate as surrounding solid tissue.

The intent of this study was not necessarily to favor the particular IC algorithm used. A PB algorithm was used because it was included in the manufacturer's standard software package. Numerous investigators have compared PB with other methods of IC. Specifically with regard to SBRT, Haedinger and colleagues compared a PB and a collapsed cone algorithm.^(7)^ To achieve the same reference PTV dose using multiple non‐coplanar or arcing 6‐MV beams, the MUs required were within 2% with either algorithm; however, differences averaged over 8% for 18‐MV beams. For three example cases where Monte Carlo calculations were performed, the PB‐based calculations were not as close to the Monte Carlo results as the collapsed cone‐based calculations.

The RTOG trial of SBRT for medically inoperable primary non‐small‐cell lung cancer (RTOG‐0236) stipulates that doses should be calculated without tissue IC, although there are plans to collect both IC‐corrected and non‐IC‐corrected dose distributions for further analysis. IC was not applied in the Indiana University trial that formed the basis of the RTOG study.^(8)^ There are two points of special importance here. First, the observation by the Indiana University group of a tendency toward higher complications for centrally located lesions might be at least partly due to the very high true doses given for the nominal doses prescribed. Second, the true doses given in the RTOG study will likely be substantially higher than the nominal prescription doses.

The concerns raised here regarding IC evoke the spirited debate between Papanikolaou and Klein: published over five years ago, the participants considered the question of whether IC should be used routinely during treatment planning for conventionally fractionated lung cancer radiotherapy.^(9)^At issue was not whether the application of IC provided a more accurate representation of the dose delivered—a matter on which the debaters agreed—but rather when IC should best be implemented universally. Papanikolaou and Klein concurred that clear physicist–physician communication would be critically important and that one of the key challenges would be the need to revisit notions of normal tissue tolerance and tumor dose‐response relationships with a more refined description of the dosimetry involved.

Given that SBRT is still in its early phase of development, there remains an opportunity to avoid the need to reanalyze dosimetric uncertainties later if adequate use of IC is applied from the start. Regarding institutional reports of lung SBRT techniques and clinical outcomes, it is particularly important to report whether or not IC was used in dose calculation and the method of IC used, if applicable. Otherwise, interinstitutional outcome comparisons will be flawed due to the possibly large uncertainty in true doses given. SBRT is an emerging treatment paradigm that involves an overall intensification of the radiation dose given in a very compact schedule. Physicists charged with establishing new SBRT programs must consider how the IC algorithm to be used compares to what others have used to ensure safety and consistent quality of treatment, regardless of which method of SBRT is selected.
